# More mentoring needed? A cross-sectional study of mentoring programs for medical students in Germany

**DOI:** 10.1186/1472-6920-11-68

**Published:** 2011-09-24

**Authors:** Felix G Meinel, Konstantinos Dimitriadis, Philip von der Borch, Sylvère Störmann, Sophie Niedermaier

**Affiliations:** 1 MeCuM-Mentor Projektbüro, Medizinische Poliklinik Campus Innenstadt, Klinikum der Ludwig-Maximilians-Universität (LMU), Pettenkofer Str. 8a, 80336 München, Germany; 2 Institut für klinische Radiologie, Klinikum der LMU, Marchioninistr. 15, 81377 München, Germany; 3 Neurologische Klinik und Poliklinik, Klinikum der LMU, Marchioninistr. 15, 81377 München, Germany; 4 Medizinische Klinik Campus Innenstadt, Klinikum der LMU, Ziemssenstr. 1, 80336 München, Germany; 5 Kinderklinik und Kinderpoliklinik im Dr. von Haunerschen Kinderspital, Klinikum der LMU, Lindwurmstr. 4, 80337 München, Germany; 6 Lehrstuhl für Didaktik und Ausbildungsforschung in der Medizin, Medizinische Klinik Campus Innenstadt, Klinikum der LMU, Ziemssenstr. 1, 80336 München, Germany

## Abstract

**Background:**

Despite increasing recognition that mentoring is essential early in medical careers, little is known about the prevalence of mentoring programs for medical students. We conducted this study to survey all medical schools in Germany regarding the prevalence of mentoring programs for medical students as well as the characteristics, goals and effectiveness of these programs.

**Methods:**

A definition of mentoring was established and program inclusion criteria were determined based on a review of the literature. The literature defined mentoring as a steady, long-lasting relationship designed to promote the mentee's overall development. We developed a questionnaire to assess key characteristics of mentoring programs: the advocated mentoring model, the number of participating mentees and mentors, funding and staff, and characteristics of mentees and mentors (e.g., level of training). In addition, the survey characterized the mentee-mentor relationship regarding the frequency of meetings, forms of communication, incentives for mentors, the mode of matching mentors and mentees, and results of program evaluations. Furthermore, participants were asked to characterize the aims of their programs. The questionnaire consisted of 34 questions total, in multiple-choice (17), numeric (7) and free-text (10) format. This questionnaire was sent to deans and medical education faculty in Germany between June and September 2009. For numeric answers, mean, median, and standard deviation were determined. For free-text items, responses were coded into categories using qualitative free text analysis.

**Results:**

We received responses from all 36 medical schools in Germany. We found that 20 out of 36 medical schools in Germany offer 22 active mentoring programs with a median of 125 and a total of 5,843 medical students (6.9 - 7.4% of all German medical students) enrolled as mentees at the time of the survey. 14 out of 22 programs (63%) have been established within the last 2 years. Six programs (27%) offer mentoring in a one-on-one setting. 18 programs (82%) feature faculty physicians as mentors. Nine programs (41%) involve students as mentors in a peer-mentoring setting. The most commonly reported goals of the mentoring programs include: establishing the mentee's professional network (13 programs, 59%), enhancement of academic performance (11 programs, 50%) and counseling students in difficulties (10 programs, 45%).

**Conclusions:**

Despite a clear upsurge of mentoring programs for German medical students over recent years, the overall availability of mentoring is still limited. The mentoring models and goals of the existing programs vary considerably. Outcome data from controlled studies are needed to compare the efficiency and effectiveness of different forms of mentoring for medical students.

## Background

Mentoring is key to a successful career in medicine [1-4]. Mentoring has shown to be essential for the acquisition of clinical and research skills, as well as career development [3-5]. Having a mentor positively correlates with productivity in research, the number of publications and grants for junior academic physicians [3]. Among medical students, having a mentor significantly increases the odds of participating in research during medical school [6]. Formal mentoring programs were found to support medical students' career planning [7-10] and enhance students' research productivity and academic orientation [11-13]. Mentoring contributes to professionalism and performance of medical students [13-16] and increases their overall well-being [8-10,17]. Mentoring also plays a significant role in supporting medical students from underrepresented minorities [16]. In addition, non-mentored graduates state that mentoring during medical school would have helped them with their residency choice and career planning [18].

Little is known about the availability and structure of mentoring programs for medical students. The most recent systematic review of the literature [19] included 14 publications describing formal mentoring programs for medical students, all from medical schools in the United States. Six of these programs offer one-on-one mentoring, 2 programs offer mentoring in small groups and the remaining 6 programs combine both forms of mentoring [19]. Most programs establish mentorship during the first 2 years of medical school, while two programs do not enroll students prior to their fourth year [19]. However, as many mentoring programs are not represented in publications, a review of the literature cannot assess the prevalence and availability of mentoring programs for medical students. It is probable that there is a lack of mentoring programs for medical students in most countries [5,19]. This shortage is likely magnified in Europe as awareness of mentoring benefits is not as developed as in the United States and larger class sizes pose a challenge for adequate mentoring [19].

In 2000, Woessner et al. [20] conducted a survey of all medical schools deans in Germany, Austria and Switzerland to determine the extent of mentoring and counseling offered to medical students. The study found that 10 (33%) medical schools offered mentoring programs with personally allocated mentors. The programs enrolled an average of 260 medical students. In addition, 8 (27%) medical schools reported offering regular career counseling. Freeman [21] criticized the survey by Woessner for being based on a vague concept of mentoring and for failing to identify the purpose of the reviewed 'mentoring' programs. Besides its methodological limitations, Woessner's survey dates back a decade. As mentoring programs are evolving rapidly in academic medicine [3,19], a study of current mentoring programs at German medical schools is well warranted.

Therefore, we sent an electronic survey to all 36 medical school deans in Germany between June and October 2009. The principal objective of our study was to attain an overview of the existing mentoring programs for medical students in Germany. We aimed to assess the prevalence of mentoring for medical students as well as to compare and categorize the structure and goals of existing mentoring programs. We further designed the study to characterize mentors, mentees, and the mentoring relationships these programs form.

## Methods

### Definition of Mentoring

The term "mentor" has been assigned a multitude of varying definitions in the literature [22-24]. Following Berk [22] and Buddeberg-Fischer [5], we defined certain basic elements as key constituents of mentoring relationships: (1) Mentoring relationships are personal in nature and involve direct interaction. (2) Mentoring relationships are long-lasting. (3) Mentoring does not merely foster an individual's skills or knowledge, but represents an integrated approach to support the individual mentee's development. This involves emotional and psychological support, direct assistance with career and professional development and role-modeling.

### Inclusion Criteria

Based on these defining characteristics we established the following inclusion criteria for programs to be included in our analysis: (1) The program is designed to include medical students as mentees, regardless of whether the program is offered to the entire university or exclusively to medical students. (2) The program establishes a relationship between a mentor and one or several mentees, or alternatively between a clearly defined number of mentors and a single mentee or a group of mentees. (3) These mentoring relationships have an intended minimum duration of one year. (4) The program is designed not merely to foster skills or knowledge, but to encourage the overall development of mentees.

### Questionnaire

We designed a questionnaire to assess key characteristics of mentoring programs, participating mentors, mentees and their relationships (See Appendices 1 & 2 for instructions to participants and the survey questionnaire). Our questionnaire solicited the following information about each program: name, web URL (if applicable), advocated mentoring model (one-on-one or group mentoring), the program's goals and intended duration, the number of participating mentees and mentors, as well as, funding and staff. We then asked about the characteristics of participating mentees (e.g., limitations to certain subgroups or certain levels of training) and mentors (levels of training, occupation). We further sought to characterize the program's mentor-mentee relationships regarding the frequency of meetings, forms of communication, incentives for mentors, the mode of matching mentors and mentees, and results of program evaluations. The questionnaire consisted of a total of 34 questions in multiple-choice (17), numeric (7) and free-text (10) format.

### Ethical Approval and Data Privacy

The LMU Medical School Ethics Committee waived ethical approval for the study. The data was collected and stored securely and analyzed anonymously.

### Electronic Survey

We distributed an electronic survey generated from our questionnaire between June and September 2009. We contacted the deans' office of all 36 medical schools in Germany by e-mail and asked them to complete our online survey or to forward the invitation to whom it concerned. If this failed to obtain a response, we contacted medical education professionals at the faculties in question through the network of Master of Medical Education graduates^23^. Additionally, we searched the webpages of all medical faculties for the terms "mentor*" and "mentoring program" to identify further contacts to interview.

### Selection of Programs

After completion of the survey, we excluded programs from the analysis that did not meet the inclusion criteria previously specified (Additional File 1, Supplementary Table S1a). For this purpose, both the reported characterization of the program and, if present, the online presentation of the program were analyzed based on content analysis following Krippendorff [25] and related to our inclusion criteria and definition of mentoring as described above. Krippendorff's concept of content analysis provides a systematic approach to analyzing textual data. It proceeds from counting keyword frequencies to analyzing their use in the specific context and coding them into categories [25].

### Data Analysis

For multiple-choice questions, we calculated the absolute number of programs and the percentage of programs that chose each particular answer. For numerical answers, the mean, median, standard deviation, as well as maximal and minimal values were determined using Microsoft Excel 2003 and SPSS version 17. For free-text questions, answer categories were derived from responses using grounded theory. Rather than using a predefined theoretical framework, grounded theory generates categories from the qualitative data [26]. As data is added, the generated categories are constantly modified and sharpened in order to optimally represent the data. Three independent researchers performed the qualitative free text analysis.

The online presentations were assessed with regard to whether they specify the goals and structure of the program and offer substantial and relevant information for potential mentees.

To categorize the evaluation results, we used Kirkpatrick's four-level outcome model for the evaluation of training programs. This model ranks outcome measures into four levels: satisfaction of participants (level 1), increase in knowledge or capability (level 2), change of behavior as a result of the learning experience (level 3), and the impact of this change in behavior (level 4) [27].

## Results

### Prevalence of formal mentoring programs for medical students in Germany

We received a total of 39 responses from the 36 medical schools in Germany, which identified a total of 25 active programs. Among the responders were 4 deans of student's affairs, 19 deans' office staff members, 11 mentoring program coordinators and 5 other medical education specialists. Disclosure of the program's budget was voluntary which resulted in missing data. Two programs were unable to specify the population size from which mentees were recruited. Four programs could not specify the number of potential mentors. Three programs did not disclose the average number of meetings between mentors and mentees per year. For all other items a complete data set was obtained.

**Figure 1:**
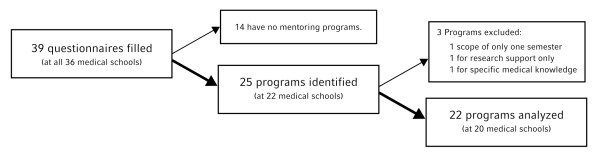
**Identification and Selection of Mentoring Programs for Medical Students in Germany**.

**Figure 2:**
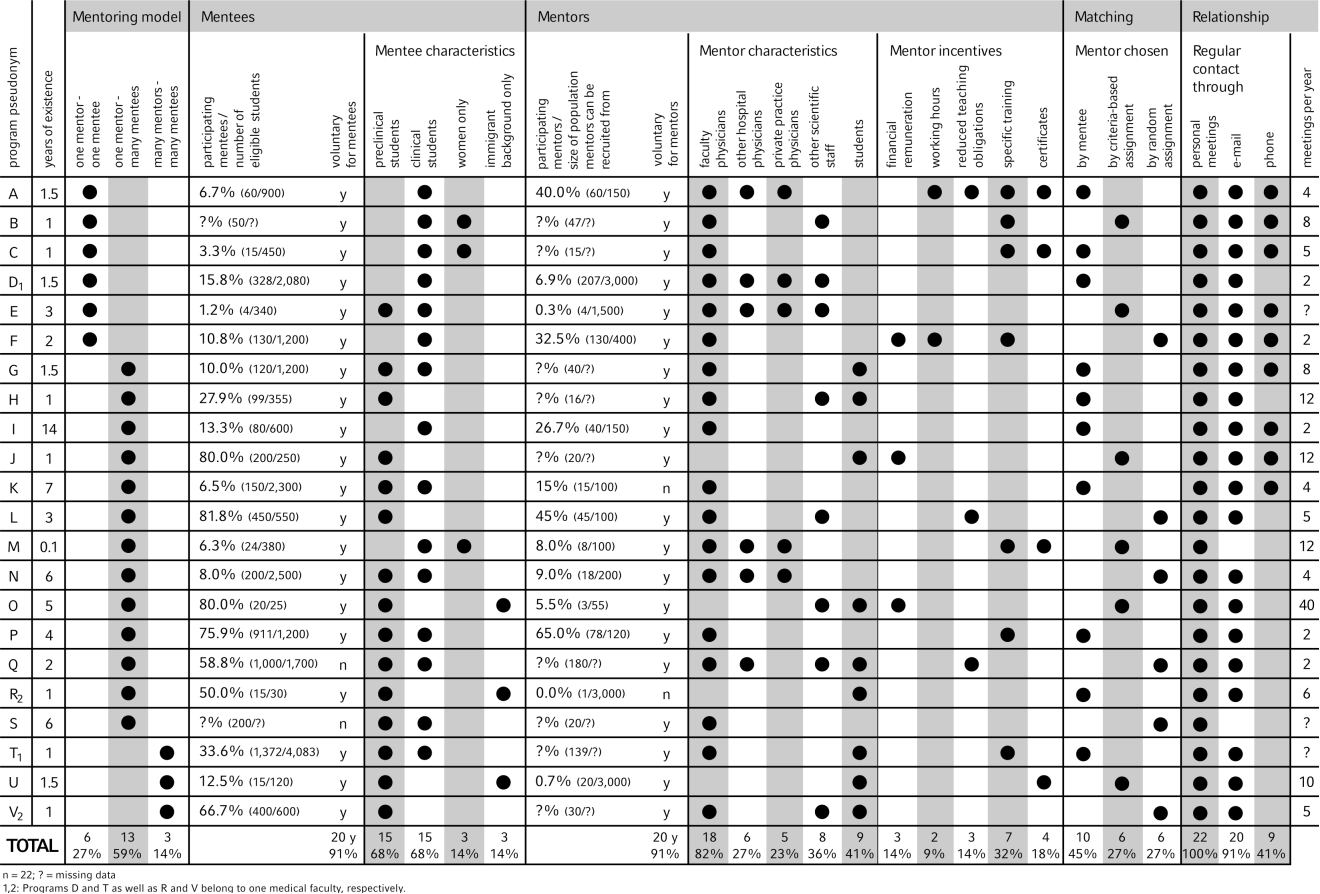
**Key Characteristics of Mentoring Programs for Medical Students in Germany**. n = 22; ? = missing data. 1, 2: Programs D and T as well as R and V belong to one medical faculty, respectively.

Three programs were excluded from our data analysis as they failed to meet certain defined inclusion criteria: one program only has an intended duration of six months, one program solely promotes mentees' research development, and one program focuses exclusively on the transfer of medical knowledge (See Figure 1 and Additional File 1, Supplementary Table S1a for programs excluded from the study). As such, of the 36 medical schools in Germany, 20 offer formal mentoring programs with 2 universities offering 2 programs (See Figure 1 and Additional File 1, Supplementary Table S1b for programs included in the study). While one program is offered to the university's entire student body, 21 programs are specifically for medical students. The 22 programs together had 5,843 medical students enrolled as mentees (Figure 2), or 6.9 to 7.4% of all German medical students [28] at the time the survey was completed (the range taking into account that mentees at the 2 universities offering 2 separate programs could partake in both). The median enrolment of mentees from the eligible student population equaled 14.6%. Interestingly, these 22 programs have only been running for a median of 1.5 years (range 0.1 - 14 years) with 14 programs being established within the last 2 years.

13 of 22 programs (59%) have a website containing information about the program. Nine of these 13 websites were found to specify the goals and structure of the program as well as provide substantial and relevant information for potential mentees.

### Goals of mentoring programs

Aspects of free-text responses were categorized into (a) declared goals of the programs, (b) means by which the program aims to achieve these goals and (c) intended character of the mentoring relationships. The majority of programs intend to build and expand mentees' networks at the medical school such as among faculty and peers (13 programs, 59%, see Table 1). For example, one program stated: "By means of mentoring, students are integrated into faculty networks which permits exchange of experiences between students and faculty." 11 programs (50%) aim to enhance students' academic performance. For instance, one participant noted that the overall goals of her school's program included "enhancing the mentee's individual learning experience and performance in medical school." Counseling students in difficulties (10 programs, 45%) was also a frequently mentioned goal, as exemplified by the following statement: "The mentor is available to mentees for specific problems they encounter and tries to help them overcome whatever hampers their success in medical school." Less commonly reported program aims include: supporting the exchange of medical knowledge (6 programs, 27%), counseling students regarding research projects including their MD thesis (6 programs, 27%) and promoting mentees' key competencies such as communication skills (4 programs, 18%). Some programs' goals include benefiting the faculty by strengthening corporate identity (4 programs, 18%), enhancing the quality of medical education at their institution (4 programs, 18%) and/or recruiting future faculty members (2 programs, 9%). A few programs cited promoting certain student groups such as increasing the presence of women in leadership positions (1 program, 5%), fostering the integration of international students (2 programs, 9%) or supporting highly gifted students (1 program, 5%) in their goals.

**Table 1:** Intended Goals of Mentoring Programs for Medical Students

Categories of mentioned goals	Number of mentions	%
Building and expanding networks	13	59
Enhancement of academic performance	11	50
Counseling students in difficulties	10	45
Exchange of medical knowledge	6	27
Counseling in research activities (thesis)	6	27
Career counseling	4	18
Promoting key competencies	4	18
Strengthening corporate identity	4	18
Enhancing quality of education	4	18
Enhancing students' satisfaction	3	14
Integration of international students	2	9
Recruiting future faculty members	2	9
Leadership development	1	5
Advancement of women	1	5
Promoting highly gifted students	1	5
**Means how to achieve these goals**	**Number of mentions**	**%**

Building a professional network	5	23
Providing a continuous contact person	4	18
Personal support	3	14
Offering an opportunity for feedback and self-reflection	1	5
**Intended character of relationships**	**Number of mentions**	%

Personal	5	23
Early in the career of mentees	4	18
On a regular basis	2	9
Long-lasting	1	5

### Strategies how programs hope to achieve their goals

Many free text answers included means by which the programs hope to achieve their goals. These included building a professional network for the mentee (5 programs, 23%, see Table 1), providing a continuous contact person (4 programs, 18%), personal support (3 programs, 14%) and offering an opportunity for feedback and self-reflection (1 program, 5%).

### Intended character of mentoring relationships

The intended characteristics of mentoring relationships within the programs were described as personal (5 programs, 23%, see Table 1), early in the career of mentees (4 programs, 18%), on a regular basis (2 programs, 9%) and long-lasting (1 program, 5%).

### Mentoring models

Six out of 22 programs (27%) offer mentoring in a one-on-one setting (Figure 2). The remaining 16 programs (73%) offer group mentoring. Among these, a single mentor supervises a group of mentees in 13 programs, whereas several mentors are responsible for a group of mentees in 3 programs. Participation is voluntary for both mentors and mentees in 18 programs (82%), whereas participation is compulsory for mentors and mentees in 2 programs (9%) each.

### Mentees

While 7 programs (32%) include medical students from all 6 curricular years, 7 programs (32%) are exclusively for preclinical year students and 6 (27%) are limited to clinical year students (Figure 2). Only female students or international/immigrant students are accepted as mentees in 3 (14%) and 2 (9%) programs, respectively.

### Mentors

The median ratio of mentees per mentor is 5.9. As expected, this ratio is significantly lower in one-on-one mentoring programs (1.0 mentees per mentor) than in group-mentoring programs (9.9 mentees per mentor). Most programs (18 programs, 82%, see Figure 2) feature faculty physicians as mentors. Fewer programs include non-faculty physicians from affiliated teaching hospitals (6 programs, 27%), physicians in private practice (5 programs, 23%), and faculty scientists (8 programs, 36%). Nine programs (41%) rely on students as mentors.

### Incentives for mentors

We also investigated whether mentoring programs offered any incentives to motivate potential mentors to participate in the program (Figure 2). Three programs (14%) pay their mentors a financial remuneration. In 2 programs (9%), mentoring activities are recognized and paid as working hours. In 3 programs (14%), faculty who engage in the mentoring program are rewarded with reduced teaching obligations. Seven programs (32%) offer specific training for mentors. Four programs (18%) issue certificates to recognize their mentors' commitment. Nine programs (41%) declared not to offer any form of incentives for mentors.

### Matching mentors and mentees

We found that 10 programs (45%) allow mentees to choose their mentors, while the remaining 12 programs (55%) assign mentors to mentees (Figure 2). Among those programs that assign mentors to mentees, 6 programs do so randomly while 6 programs take specific criteria into account, such as areas of professional interest, preference for research versus clinical careers and personal characteristics. Among the 10 programs in which mentees choose their mentors, 8 programs offer auxiliary resources to support informed choice. These include online mentor profiles in 7 programs, paper-based mentor profiles in one program and personal interviews with mentors and mentees in 2 programs. One program offers regular get-together events to acquaint mentees with potential mentors.

### Means of communication

Personal meetings between mentors and mentees are a universal element of all 22 mentoring programs (Figure 2). On average, mentors and mentees meet 7 times per year (median 5, range 2 - 40). 20 programs (91%) reported that e-mail is used regularly as a form of communication between mentors and mentees. Telephone is a regular form of communication in 9 programs (41%). No other means of communication are used regularly.

### One-on-one mentoring

Six programs offer one-on-one mentoring to medical students in Germany. Four of them are limited to students in their clinical years and 2 of these are exclusively for female students. On average, 98 students are enrolled as mentees in each of these programs (median 55, range 4 - 328). In total, at the time of the survey 587 medical students (0.7% of all German medical students) are enrolled in one-on-one mentoring programs with more than half of them (n = 328; 56%) in one program. In the one-on-one structure, personal meetings between mentors and mentees take place an average of 4 times per year (median 3, range 2-8). Three one-on-one programs allow mentees to choose their mentor (2 of which offer online mentor profiles), while 3 programs assign mentors to their mentees (2 of which use profile questionnaires for matching mentors and mentees).

### Funding and staff

Eleven programs (50%) reported receiving funding from their respective university. Eight programs (36%) are funded by tuition fees and 5 programs (23%) by third party funds. Nine programs (41%) employ an average of 1.1 physicians or scientific staff members to administrate the mentoring program. Student assistants run 11 programs (50%). Five programs (23%) employ a secretary or other non-scientific member of staff. Additionally, we asked participants to voluntarily provide their program's annual budget. Several programs did not provide this information. For some who did, the budget seems implausibly low relative to the employed staff. Therefore, the budget required to run mentoring programs for medical students cannot be legitimately analyzed from our data set.

### Evaluation and outcome data

15 programs (68%) reported conducting evaluations on a regular basis. Two programs reported that results had been submitted for publication in a scientific journal. Evaluations are performed through online or paper surveys, interviews or feedback meetings. Most evaluations focused on mentee and mentor satisfaction corresponding to Kirkpatrick's level 1. Four programs evaluated topics discussed between mentors and mentees and what mentees perceived as the impact of their relationship (Kirkpatrick's level 2). One program found that geographical distance had no impact on the quality of the mentoring relationship. None of the evaluations measured the efficacy of mentoring in altering behavior or achieving objective outcome criteria (Kirkpatrick's levels 3 & 4).

## Discussion

### Emergence of mentoring programs

Our data show that there has been a dynamic emergence of mentoring programs for medical students over recent years in Germany. Out of 22 mentoring programs with personally allocated mentors at German medical schools, 14 have been established within the last 2 years. Despite methodological differences, it is remarkable that in the study of Woessner et al. in 2000 [20] only 10 German medical schools offered such programs. Only one program persisted over the past 10 years (Figure 2, Program I). This may reflect an international trend towards increased awareness of the benefits of mentoring in medical education [3,19]. However, the creation of numerous mentoring programs at German medical schools may also be due to the introduction of tuition fees in Germany. After the German Federal Court ruled to lift the ban on university tuition fees in 2005, 21 of the 36 medical universities introduced tuition fees. These are intended to improve study conditions and can be used to fund extracurricular programs. Indeed, 8 of the 22 programs in our study reported receiving funding through tuition fees. As states may drop tuition fees for political reasons in the near future, it remains unclear how future funding will be covered.

### Diversity of goals

Although we used clearly defined inclusion criteria, we found mentoring programs with diverse declared goals. This heterogeneity reflects the multifaceted notion of mentoring. In a recent review on mentoring programs for medical students by Frei et al. [19], the programs' goals were classified into four categories: (1) providing career counseling, (2) developing professionalism and supporting personal growth, (3) recruiting students into research and academic medicine, and (4) attracting students into specific disciplines. Most of the categories generated by our data correspond well with aspects of Frei's categories. Interestingly however, one of the most frequently mentioned goals in our study, the enhancement of academic performance, is not covered by the classification presented by Frei et al.

### Measuring the effectiveness of mentoring programs

The mentoring programs in our study represent various forms of mentoring ranging from one-on-one to group mentoring, and from peer-mentoring to mentoring by senior faculty members. To establish characteristics of successful mentoring programs, it would be necessary to correlate the structures of various mentoring programs with their outcome. However, as outlined above, the outcome levels on which the programs in our study are evaluated are insufficient to establish this correlation. Evaluations of mentoring programs published in the literature are also rarely based on validated questionnaires [3,19]. Therefore, further research is needed to measure the effectiveness and efficiency of mentoring for medical students and establish factors influencing the success of mentoring programs. To our knowledge, only one mentoring program has been evaluated in a randomized controlled study design [13]. In this study, medical students at the University of California Los Angeles who were enrolled in a mentoring program reported an increased satisfaction with clinical and scholarly experiences during their final year and felt better prepared for residency [13].

One of the reasons for this lack of research certainly lies in the difficulties associated with measuring the effectiveness of mentoring in medicine. Currently available instruments for the evaluation of formal mentoring programs in medicine, such as the tools developed by Morzinski et al. [29] or Berk et al.[22], are tailored for junior faculty and some of their criteria are not commonly applicable to medical students. Distinct tools that measure the outcome as well as assess the process of mentoring for medical students need to be developed.

Besides focusing on the benefits for student mentees, the evaluation of medical student mentoring programs should also address the benefits for mentors and the educational institution. It has been shown recently that mentoring medical students led to personal and professional development of the mentoring physician [30]. By offering mentoring programs, medical schools could be able to attract high-potential students and tie successful graduates to the university. If mentoring can make medical education more effective, its benefits might ultimately be traced to improved patient care. Importantly, further research into mentoring for medical students should not ignore potential problems and difficulties such as conflict between the mentoring and supervisory roles of mentors, confidentiality breaches, mentor bias and role confusion [31,32]. These issues should be carefully considered when drawing conclusions about how to set up a successful mentoring program [33]. These difficulties in measuring the effectiveness of mentoring may be the reasons why our study did not obtain evaluation datasets substantial enough to analyze them validly and draw conclusions for the optimal characteristics of a mentoring program for medical students.

### Is there a lack of mentoring for medical students?

It has been hypothesized in the literature that there is a lack of mentoring for medical students in most countries [5,19]. Indeed, our data shows that only a very limited number of medical students (6.9 - 7.4% of all German medical students) are enrolled in formal mentoring programs and a much smaller fraction (0.7%) receive one-on-one mentoring at one point in time. Considering that many of the included programs do not form mentoring relationships designed to last throughout the mentee's career in medical school, the percentage of medical students who enroll in formal mentoring programs at some point during medical school is likely to be significantly higher.

Our study has assessed the prevalence of formal mentoring programs for medical students in Germany. However, for two main reasons, only limited conclusions can be drawn from this. Firstly, the enrolment rates differed greatly between programs. This raises the question whether all programs were truly available to all eligible students. Differences in publicity of the program, recruitment and selection of mentees may account for different enrollment rates. These factors were not sufficiently measured by our survey and need to be taken into account when assessing the true availability of the programs in our study to the eligible population of students. Secondly, students might form satisfying mentoring relationships outside or in the absence of formal mentoring programs or might simply not desire mentoring.

However, our data does provide some indirect evidence that there is an unmet demand for mentoring among medical students. Firstly, 16 out of 36 medical schools do not offer any mentoring program for medical students to date. Considering evidence that medical students encounter severe difficulties finding a suitable mentor outside formal programs [34], it seems likely that at least these institutions will have students without access to a mentor. Secondly, our survey identified a one-on-one mentoring program that was able to attract 328 students (15.8% of eligible students) for one-on-one mentoring within 1.5 years after the launch of the program (see Figure 2, program D). This indicates that medical students at this institution had a significant demand for mentoring which was not met by informal mentoring relationships.

## Conclusions

To our knowledge, there are no similar studies that could be used as a reference for international comparison. Meta-analyses of the literature [5,19] cannot serve to assess the prevalence of mentoring programs given our finding that none of the mentoring programs in our study had been published in journal articles.

In conclusion, our study assessed the availability of mentoring programs for medical students in Germany. We found that despite the emergence of numerous programs over the past few years only a limited number of medical students are enrolled in formal mentoring programs and only a small percentage of those receive mentoring in a one-on-one mentoring setting.

To our knowledge, our survey instrument represents the first detailed questionnaire developed for a cross-sectional study of mentoring programs covering key characteristics of formal mentoring programs. Elements of our survey may be used to develop a standardized survey instrument that will need to be validated to be used in further national or international studies.

Higher-level outcomes of mentoring programs as well as the quality of the mentoring process need to be assessed with validated instruments in order to appraise their long-term impact on medical students' professional development and behavior. Based on this data, mentoring programs for medical students could be improved and managed to maximize their benefit for mentees, mentors and medical schools.

## Competing interests

The authors declare that they have no competing interests.

## Authors' contributions

FGM participated in the conception and design of the study and questionnaire, carried out the data acquisition, participated in the analysis and interpretation of data and wrote the manuscript. KD participated in the conception and design of the study and questionnaire, participated in the analysis and interpretation of data and critically reviewed the manuscript. PvdB participated in the conception and design of the study and questionnaire, participated in the analysis and interpretation of data, prepared the figures and critically reviewed the manuscript. SS participated in the conception and design of the study and questionnaire, participated in the analysis and interpretation of data and critically reviewed the manuscript. SN participated in the analysis and interpretation of data and critically reviewed the manuscript. MRF participated in the conception and design of the study and questionnaire, participated in the analysis and interpretation of data and critically reviewed the manuscript. All authors read and approved the final manuscript.

## Appendix 1: Instructions to Participants

Dear Sir or Madam,

You have been referenced as a contact person for a mentoring program at your university. We are conducting a study about mentoring programs for medical students at German medical schools. The study will provide an overview of existing mentoring programs for medical students in Germany. We would like to ask you to participate in our study. You can complete our survey online by clicking on the link provided below. Completing the questionnaire will take you approximately 15 minutes. You can save your answers at any time and proceed later. If you prefer to answer our questions on the phone, please let us know.

All mentoring programs that enroll medical students as mentees should be included into the study. By mentoring we understand a steady relationship between a mentor and one or several mentees, or alternatively between a clearly defined number of mentors and a single mentee or a group of mentees designed to foster the development of mentees. By mentoring we do not understand tutoring (designed to communicate factual knowledge), career advising offices which do not establish a steady mentor-mentee-relationship or supervision for specific (eg, scientific) projects.

If your university offers more than one mentoring program that involve medical students as mentees, simply complete the questionnaire multiple times. If any questions arise during completion of our survey, please do not hesitate to contact us.

Yours sincerely,

## Appendix 2: Survey Questionnaire

### 1 The Institution

1.1 Which university do you respond for? [FREE TEXT]

1.2 At your university, is there a mentoring program that involves medical students as mentees? [YES/NO] (if 'no', skip to 6.1)

1.3 What is the name of the mentoring program? [FREE TEXT]

1.4 Is the mentoring program designed exclusively for students at the faculty of medicine or the entire university? [MC: MEDICAL FACULTY/ENTIRE UNIVERSITY/OTHER]

1.5 Is there a website that offers information about your program? If yes, please provide URL. [FREE TEXT]

### 2 Key Characteristics of the Mentoring Program

1.6 What are the goals of your mentoring program? [FREE TEXT]

1.7 Please specify the ratio of mentors and mentees in a typical interaction within your program (not the overall ratio). [MC: one mentor - one mentee (individual mentoring)/one mentor - multiple mentees (group mentoring)/multiple mentors - one mentee (individual mentoring, e.g. tandem model)/multiple mentors - multiple mentees (group mentoring)/other]

1.8 How long has your program been running (in years)? [NUMERICAL]

1.9 How is your mentoring program funded? [MC: third party funding/university funds/tuition fees/other]

1.10 What is the annual budget of your mentoring program? [numerical]

1.11 How much staff is employed primarily for the administration of the mentoring program? [scientific staff/physicians [numerical]/secretaries [numerical]/student assistants [numerical]/other [numerical]]

### 3 The Mentoring Relationships

1.12 Is the participation in your program voluntary for mentees? [YES/NO]

1.13 Is the participation in your program voluntary for mentors? [YES/NO]

1.14 How many mentees and mentors currently participate in your mentoring program? [NUMBER OF ACTIVE MENTEES IN THE PROGRAM [NUMERICAL]/NUMBER OF ACTIVE MENTORS IN THE PROGRAM [NUMERICAL]/SIZE OF POPULATION FROM WHICH MENTEES ARE RECRUITED (INCLUDING PARTICIPATING MENTEES) [NUMERICAL]/SIZE OF POPULATION FROM WHICH MENTORS ARE RECRUITED (INCLUDING PARTICIPATING MENTORS) [NUMERICAL]]

1.15 What is the mentees' educational level? [ALL STUDENTS/STUDENTS OF PRECLINICAL YEARS ONLY/STUDENTS OF CLINICAL YEARS ONLY/OTHER]

1.16 Is the mentoring program designed exclusively for a specific subgroup of students? [NO/FEMALE STUDENTS ONLY/STUDENTS WITH FOREIGN BACKGROUND ONLY/OTHER]

1.17 What is the mentors' professional profile? [ACADEMIC PHYSICIANS/PHYSICIANS AT NON-ACADEMIC HOSPITALS/PHYSICIANS IN PRIVATE PRACTICE/NON-PHYSICIAN UNIVERSITY SCIENTISTS/SCIENTISTS OUTSIDE THE UNIVERSITY/OTHER]

1.18 What is the mentors' educational level? [STUDENTS (PEER MENTORING)/RESIDENT PHYSICIANS OR RECENT GRADUATES/ATTENDING PHYSICIANS OR MULTIPLE YEARS OF WORK EXPERIENCE/PROFESSORS/OTHER]

1.19 How many meetings between mentors and mentees are intended by the program and how many actually take place annually? [NUMBER OF INTENDED MEETINGS [NUMERICAL]/NUMBER OF ACTUAL MEETINGS [NUMERICAL]]

1.20 Which forms of communications are regularly used between mentors and mentees? [PERSONAL MEETINGS/EMAIL/PHONE/OTHER]

1.21 Which topics are typically discussed between mentors and mentees? [FREE TEXT]

1.22 Are there any extrinsic incentives for becoming a mentor in your program? [NONE/FINANCIAL COMPENSATION/RECOGNITION AS WORKING HOURS/REDUCED TEACHING OBLIGATIONS/SPECIFIC TRAINING FOR MENTORS/GREATER CHANCE OF PROMOTION/CERTIFICATES/OTHER] (skip to 4.1 unless "financial compensation" is ticked)

1.23 What is the financial compensation for mentors (in euro per mentee and year) [NUMERICAL]

### 4 Matching Mentors and Mentees

1.24 How are mentors and mentees matched? [MC: MATCHED BY PROGRAM/MENTEE CHOOSES MENTOR/MENTOR CHOOSES MENTEE/OTHER]

1.25 (only if 4.1 is "mentee chooses mentor") Does the mentee choose his/her mentor from a preselected group of potential mentors (mentors recruited by program) or from the entire population of potential mentors (mentors recruited by mentee)? [PRESELECTED GROUP/ENTIRE POPULATION]

1.26 (only if 4.1 is "mentee chooses mentor") Are there any auxiliary resources for the selection of a mentor by the mentee? [MC: NONE/PAPER-BASED MENTOR PROFILES/ONLINE MENTOR PROFILES/MENTORING-SPECIFIC GET-TOGETHER EVENTS/PERSONAL INTERVIEW OF MENTEES AND/OR MENTORS BY THE PROGRAM/OTHER]

1.27 (only if 4.1 is "mentor chooses mentee") Are there any auxiliary resources for the selection of a mentee by the mentor? [MC: NONE/PAPER MENTEE PROFILES/ONLINE MENTEE PROFILES/MENTORING-SPECIFIC GET-TOGETHER EVENTS/PERSONAL INTERVIEW OF MENTEES AND/OR MENTORS BY THE PROGRAM/OTHER]

1.28 (only if 4.1 is "matched by program") How is the matching performed? [MC: RANDOMLY/TAKING SPECIFIC CRITERIA INTO ACCOUNT]

1.29 (only if 4.5 is "specific criteria") Which criteria are used to match mentors and mentees? [FREE TEXT]

1.30 How are mentors recruited for the program? [FREE TEXT]

### 5 Evaluation

1.31 Is an evaluation of the mentoring relationships performed? [YES/NO] (if "no", skip to 5.5)

1.32 How is the evaluation performed? [FREE TEXT]

1.33 What are key results of your evaluation? [FREE TEXT]

1.34 Have results of your evaluation been published? If so, where? [YES/NO + FREE TEXT COMMENT]

1.35 Besides the mentoring relationships, does your mentoring program include special events (e. g. lectures, seminars, excursions etc.)? If so, please describe. [FREE TEXT]

### 6 Concluding questions

1.36 Please provide your name for further questions. [FREE TEXT]

1.37 Please provide your phone number and/or e-mail address. [FREE TEXT]

1.38 Would you like to be acknowledged in a publication of this study? [FREE TEXT]

## Pre-publication history

The pre-publication history for this paper can be accessed here:

http://www.biomedcentral.com/1472-6920/11/68/prepub

## Supplementary Material

Additional file 1 **Supplementary table S1**. Supplementary table S1 lists the names and institutions of all programs excluded from (1a) and included in the study (1b). Click here for file
