# Supplementary material for: More mentoring needed? A cross-sectional study of mentoring programs for medical students in Germany
Source: BMC Med Educ. 2011 Sep 24;11:68. doi: 10.1186/1472-6920-11-68 (PMC3191506; doi:10.1186/1472-6920-11-68)
Supplement: Additional file 1 — Supplementary table S1. Supplementary table S1 lists the names and institutions of all programs excluded from (1a) and included in the study (1b). [file 1472-6920-11-68-S1.DOC]

**Supplementary Table S1**

**Supplementary Table S1a. Mentoring programs excluded from the study**

| **Name of University / Medical School** | **Name of Program** | **Reason for Exclusion** |
| --- | --- | --- |
| Ernst-Moritz-Arndt-Universität Greifswald | Gerhard-Domagk-Nachwuchsförderprogramm | Exclusively research-oriented |
| Eberhard Karls Universität Tübingen | Mentorenprogramm Medizin | Mentoring relationships designed to last only one semester |
| Albert-Ludwigs-Universität Freiburg | Mentorenprogramm -Wahlfach Vorklinik | Designed to transfer specific medical knowledge |

**Supplementary Table S1b. Mentoring programs included into the study**

| **Name of University / Medical School** | **Name of Program** | **URL (when available)** |
| --- | --- | --- |
| **One-on-one mentoring programs** |  |  |
| Rheinisch-Westfälische Technische Hochschule Aachen | TANDEMmed | www.tandemmed.ukaachen.de |
| Friedrich-Alexander-Universität Erlangen-Nürnberg | ARIADNEmed Mentoring Programm | www.mentoring.med.uni-erlangen.de |
| Ruprecht-Karls-Universität Heidelberg | Mentoring im Mentoren-Tutoren-Programm | www.mentoren-tutoren-programm.de |
| Ludwig-Maximilians-Universität München | MeCuM-Mentor (one-on-one-Teil) | www.mecum-mentor.de |
| Technische Universität München | TUM Mentorenprogramm Medizin | www.meditum.de |
| Universität Witten/Herdecke | Wittener Tandem | www.uni-wh.de/netzwerk/karrierebegleitung/ mentoring/wittener-tandem/ |
| **Group mentoring programs** | | |
| Universität Ulm | Mentorateprogramm der Medizinischen Fakultät | www.uni-ulm.de/med/fakultaet/ studium-und-lehre/ulmer-lehr-und-lernkonzept/mentorateprogramm.html |
| Universität Hamburg | Mentorenprogramm |  |
| Ruhr-Universität Bochum | Patenschaftsprogramm |  |
| Universität des Saarlandes, Medizinische Fakultät Homburg | Mentorenprogramm |  |
| Philipps-Universität Marburg | Mentorenprogramm |  |
| Ruprecht-Karls-Universität Heidelberg, Medizinische Fakultät Mannheim | Mentorenprogramm MaReCuM | www.umm.uni-heidelberg.de/studium/ studma/marecum/grundstudium.html |
| Julius-Maximilians-Universität Würzburg | Mentoring studmed | www.uni-wuerzburg.de/ueber/fakultaeten/ medizin/studium_und_lehre/mentoring/ |
| Universität zu Köln | Mentorenprogramm | www.medfak.uni-koeln.de/153.html |
| Otto-von-Guericke-Universität Magdeburg | Ausländer-Tutorien |  |
| Universität zu Lübeck | Das Mentorenprogramm der Medizinischen Fakultät | www.medizin.uni-luebeck.de |
| Westfälische Wilhelms-Universität Münster | OMENTUM | www.omentum.ms |
| Technische Universität Dresden | Tutorium für ausländische Studierende |  |
| Justus-Liebig-Universität Gießen | Hochschulmentoren |  |
| Ludwig-Maximilians-Universität München | MeCuM-Mentor (peer-to-peer Teil) | www.mecum-mentor.de |
| Charité Universitätsmedizin Berlin | OFIS (Orientierung für internationale Studierende) Mentoring | www.charite.de/studium/international/ ofis/mentoring |
| Technische Universität Dresden | Konzept zur Förderung der Zusammenarbeit und Identifikation der Studierenden mit der Med. Fakultät Carl Gustav Carus der TU Dresden | www.fsmed-dresden.de |
